# Can severe aortic stenosis be identified by emergency physicians when interpreting a simplified two-view echocardiogram obtained by trained echocardiographers?

**DOI:** 10.1186/s13089-015-0022-8

**Published:** 2015-04-18

**Authors:** Hasan Alzahrani, Michael Y Woo, Chris Johnson, Paul Pageau, Scott Millington, Venkatesh Thiruganasambandamoorthy

**Affiliations:** Department of Emergency Medicine, University of Ottawa, 1053 Carling Avenue, Ottawa, ON K1Y 4E9 Canada; Division of Cardiology, University of Ottawa, 40 Ruskin Street, Ottawa, Ontario K1Y 4W7 Canada; Division of Critical Care, University of Ottawa, 1053 Carling Avenue, Ottawa, ON K1Y 4E9 Canada; Department of Epidemiology and Community Medicine, University of Ottawa, 451 Smyth Road, Ottawa, Ontario K1H 8M5 Canada; The Ottawa Hospital Research Institute, 40 Ruskin Street, Ottawa, Ontario K1Y 4W7 Canada

**Keywords:** Aortic stenosis, Echocardiography, Point-of-care ultrasonography, FOCUS

## Abstract

**Background:**

Aortic stenosis (AS) is a common valve problem that causes significant morbidity and mortality. The goal of this study was to determine whether an emergency physician (EP) could determine severe AS by reviewing only two B-mode echocardiographic views (parasternal long axis (PSLA) and parasternal short axis (PSSA)) obtained by trained echocardiographers.

**Methods:**

A convenience sample of 60 patients with no AS, mild/moderate AS or severe AS was selected for health record and echocardiogram review. The echocardiograms were performed in an accredited echocardiography laboratory. An EP blinded to the cardiologist’s final report reviewed the PSLA and PSSA views after the cases were randomly sorted. Severe AS was defined as no cusp movement seen by the EP reviewers. A second EP independently reviewed 25% of randomly selected patients for inter-rater reliability. Collected data included patient demographics, EP interpretation and details of each echo view (quality, the number of cusps visualized, presence of calcification) and compared to final cardiology reports. Analyses included descriptive statistics, test characteristics for severe AS and kappa for agreement.

**Results:**

The mean age was 75.3 years (range 18 to 90) with 36.7% females. The cardiologist’s diagnosis was as follows: 38.3% severe AS, 28.3% mild/moderate AS and 33.3% no AS. The PSSA view was poorer in quality compared with the PSLA (33.3% vs. 13.3%, *p* = 0.02), but the PSSA view was better than PSLA to visualize all three cusps (83.3% vs. 0%, *p* = 0.001). There was no difference in the presence of calcification between the mild/moderate and severe AS groups (94.1% vs. 100.0%, *p* = 0.46). The sensitivity and specificity for EP diagnosis of severe AS was 75.0% (95% CI 56.7% to 85.4%) and 92.5% (83.3% to 97.7%). The kappa for severe AS was 0.69 (0.41 to 0.85), and there was no significant difference between observers in the quality of the view, presence of aortic calcification and the number of cusps visible.

**Conclusions:**

PSLA and PSSA views obtained by trained echocardiographers can be interpreted by an EP with appropriate training to identify severe AS with good specificity. Further larger prospective studies are required before widespread use by EPs.

**Electronic supplementary material:**

The online version of this article (doi:10.1186/s13089-015-0022-8) contains supplementary material, which is available to authorized users.

## Background

Aortic stenosis (AS) is a common valve problem especially in patients older than 65 years of age [1]. The definition of severe aortic stenosis is a valve area small enough to cause the symptoms of aortic stenosis that increases the risk of sudden death resulting in the need for urgent aortic valve replacement [2]. The presence of severe AS carries a 50% increase in risk of myocardial infarction and cardiovascular death compared to the normal population [3]. After onset of symptoms in severe AS, average survival is 2 to 3 years with a high risk of sudden death if left untreated [4]. Patients with AS can present with different symptoms depending on the severity of stenosis ranging from chest pain, syncope, heart failure and/or sudden cardiac death. This has been observed in 0% to 5% of asymptomatic patients and in 8% to 34% of symptomatic patients [4-6]. The diagnosis of severe AS as a cause of a patient’s chest pain, dyspnea or syncope is challenging. Transthoracic echocardiography is the standard means for evaluation of aortic stenosis severity [7,8]. This comprehensive test takes about 30 min to perform involving more than five views and the use of B-mode, colour and spectral Doppler. In addition, access to the test on an urgent basis is limited in the emergency department (ED).

Normally, the aortic valve has three principle components: the annulus, three cusps and three commissures. Anatomical variants do exist and the aortic valve may be unicuspid, bicuspid or quadricuspid. Cowie and Kluger indicated that qualitative and limited quantitative assessment of the peak velocity allows determination of the presence of aortic stenosis [9]. Another report indicates that if there is any single valve movement then there is no severe AS [10]. The objective of this study was to determine whether emergency physicians (EP) could interpret severe AS on a qualitative review of only the parasternal long axis (PSLA) and parasternal short axis (PSSA) B-mode echocardiography images obtained by trained echocardiographers when compared with cardiology interpretation of the complete comprehensive echocardiogram. This could decrease the technical expertise and time required to perform this test. EPs could then potentially use this simplified approach to identify and screen for severe AS that may need admission to hospital and allow earlier intervention to address this serious problem.

## Methods

### Study design

The study design was a retrospective case control health records review of a convenience sample of patients who had an echocardiogram (Additional file 1). The study was approved by the Ottawa Health Science Network Research Ethics Board.

### Study setting and population

Patients with known varying degrees of aortic stenosis (normal, mild/moderate and severe AS - Additional files 2, 3, 4, 5, 6 and 7) diagnosed at an accredited echocardiography laboratory were identified and anonymized by a cardiologist.

### Study protocol

A convenience sample of 60 known cases of varying degrees of aortic stenosis was identified from an accredited echocardiography laboratory performed by trained echocardiographers. Inclusion criteria were adults over 18 years old and any patients that had a cardiology performed echocardiography study for angina, syncope, heart failure symptoms or murmur. Cases were excluded if the patients were known to have an aortic valve replacement or any other congenital cardiac abnormality including bicuspid aortic valve. All echocardiograms were then anonymized and randomized by a cardiologist. The EP reviewer had received 1 month of limited echocardiography training in an accredited laboratory that involved the performance and interpretation of approximately 50 comprehensive echocardiograms. This single EP reviewer was blinded to all clinical information and only reviewed the PSLA and the PSSA views. The EP reviewer used qualitative assessments only of ejection fraction, aortic valve cusp morphology including the quality of the views based on the experience of the EP reviewers, degree of calcification and assessment of cusp mobility to judge aortic stenosis severity. Reviewers defined severe AS as the absence of any cusp movement. Based on reviewing aortic valve morphology using only these two views, the EP completed a standardized case record form and determined if there was no AS, mild/moderate AS, or severe AS. This was then compared to the formal cardiology echocardiography report to determine the accuracy of the blinded two-view assessment by the EP. Another EP randomly selected 25% of the cases for review to determine validity and inter-rater reliability.

### Data analysis

Descriptive statistics with means and range were calculated. Microsoft Excel data sheets were exported into Statistical Analysis System (SAS) for data analysis. Sensitivity and specificity were calculated for assessing severe AS test characteristics. Kappa values were calculated to determine the inter-observer reliability between EP reviewers for the diagnosis of severe AS.

## Results

The mean age was 75.3 years (range 18 to 90) with 36.7% females. Based on the cardiologist’s interpretation of the echocardiograms performed at the accredited echocardiography laboratory, the cases had the following distribution: 38.3% (23/60) severe AS, 28.3% (17/60) mild or moderate AS and 33.3% (20/60) no AS. Ten patients were identified by cardiology to have an ejection fraction of less than 45%.

Although the PSSA was poorer in image quality compared with the PSLA (33.3% vs. 13.3%, *p* = 0.02), the PSSA permitted visualization of all three cusps compared with the PSLA (83.3% vs. 0%, *p* = 0.001). There was no difference in the presence of calcification between the mild/moderate and severe AS groups (94.1% vs. 100.0%, *p* = 0.46). The sensitivity and specificity for EP diagnosis of severe AS was 75.0% (95% CI 56.7% to 85.4%) and 92.5% (83.3% to 97.7%). The kappa for severe AS was 0.69 (0.41 to 0.85), and there was no significant difference between observers in the quality of the view, presence of aortic calcification and the number of cusps visible. Three patients were identified by cardiology as having no AS when the EP felt there was severe AS. One of those patients had an ejection fraction of less than 45%. There were seven patients identified by cardiology as having severe AS when the EP felt there was no severe AS. Six of the seven patients had calcification of the aortic valves and three of the seven patients had poor quality views.

## Discussion

The objective of this study was to determine whether emergency physicians who have specific training in ultrasonography could identify severe AS on review of only the PSLA and PSSA B-mode echocardiography images. Based on this study, when severe AS on echocardiography is defined as absence of any cusp movement, EPs with ultrasonography training are able to identify severe AS with high specificity and moderate sensitivity compared with cardiology interpretations. The strong kappa values support good inter-rater reliability between EPs when recognizing severe AS. This is the first known study of this kind.

Although this study showed excellent specificity, in emergency settings, higher sensitivity is desirable to help rule out a disease. Higher sensitivity could be obtained at the expense of specificity if severe AS were defined as single cusp movement as opposed to no cusp movement.

Current recommendations to determine AS require comprehensive echocardiograms using multiple views, B-mode, colour and spectral Doppler [11,12]. Assessment of left ventricular function is important in the assessment of aortic stenosis due to its effect on transvalvular gradient [13]. In this study, there was one case where a low ejection fraction may have resulted in the EP interpreting severe AS using only the two B-mode views while the cardiologist interpretation of the full echocardiogram identified no severe AS. Prior studies demonstrate that EPs are able to acquire and interpret assessment of left ventricular function [14]. Other factors that may have contributed to the missed cases of severe AS include poor quality views and calcification of the aortic valves. Valvular assessment and in particular identifying severe aortic stenosis is not currently part of the Focus Cardiac Ultrasound (FOCUS) exam [15,16].

Performing only the PSLA and PSSA B-mode views is faster than performing comprehensive echocardiograms and point-of-care providers already have the skills to perform and interpret these FOCUS exams.

Further larger prospective studies to determine the use of FOCUS by point of care providers to determine severe AS is required to demonstrate the potential to improve this diagnosis in a timely fashion.

### Limitations

The small sample size of this pilot study limits the generalizability of the results. Given the retrospective design, the results should be used for further hypothesis generating only at this time. It is important to note that EPs did not perform the echocardiogram but rather reviewed the images only, and this study has not explored the ability of EPs to acquire the PSLA and PSSA views of the aortic valve. A single EP reviewed the images and this may not be generalizable to all EPs although good correlation was obtained with another EP reviewer. The convenience sample included only tri-leaflet aortic valves and so the results are not generalizable to patients with bicuspid valves or other congenital aortic valve abnormalities.

## Conclusions

This study demonstrates that a simplified two-view B-mode PSLA and PSSA echocardiogram obtained by trained echocardiographers and interpreted by an EP with appropriate training can identify severe AS with good specificity. Further larger prospective studies are required to evaluate the accuracy of identifying severe AS by EPs performing FOCUS.

## Additional files

Additional file 1:
**Standardized case record form.** Data collection form. Additional file 2:
**Cine clip of normal aortic valves in the PSSA view.** PSSA view of a patient with no aortic stenosis on comprehensive echocardiography. Note that the aortic valve leaflets are thin with no calcification. In the parasternal short axis view, all three aortic valve cusps are visualized and all three have normal cusp excursion. Additional file 3:
**Cine clip of normal aortic valves in the PSLA view.** PSLA view of a patient with no aortic stenosis on comprehensive echocardiography. Note that the aortic valve leaflets are thin with no calcification. In the parasternal long axis view, two aortic valve cusps can be visualized, and each has normal cusp opening. Additional file 4:
**Cine clip of non severe aortic stenosis in the PSSA view.** PSSA view of a patient with moderate aortic valve stenosis on comprehensive echocardiography. The parasternal short axis view permits visualization of all three aortic valve cusps, two of which are heavily calcified. Again, the right coronary cusp (upper most cusp) has normal excursion, suggesting that the degree of aortic stenosis is unlikely to be severe. Additional file 5:
**Cine clip of non severe aortic stenosis in the PSLA view.** PSLA view with moderate aortic valve stenosis on comprehensive echocardiography. Note that in the parasternal long axis view, two aortic valve cusps are visualized. One of the cusps has heavy calcification indicating some degree of aortic valve stenosis may be present. However, this cusp has normal excursion, suggesting that severe aortic stenosis is unlikely. Additional file 6:
**Cine clip of severe aortic stenosis in the PSSA view.** PSSA view of a patient with severe aortic stenosis on comprehensive echocardiograpy. The short axis view permits visualization of all three aortic valve cusps, none of which has normal cusps excursion, highly suggestive of severe aortic valve stenosis. Additional file 7:
**Cine clip of severe aortic stenosis in the PSLA view.** PSLA view of a patient with severe aortic stenosis on comprehensive echocardiograpy. Note that in the parasternal long axis view, two aortic valve cusps are visualized and each is heavily calcified. Very limited cusp excursion of both cusps is concerning for severe aortic stenosis.
