# Supplementary material for: Can severe aortic stenosis be identified by emergency physicians when interpreting a simplified two-view echocardiogram obtained by trained echocardiographers?
Source: Crit Ultrasound J. 2015 Apr 18;7:5. doi: 10.1186/s13089-015-0022-8 (PMC4409610; doi:10.1186/s13089-015-0022-8)
Supplement: Additional file 1: — Standardized case record form. Data collection form. [file 13089_2015_22_MOESM1_ESM.docx]

**Study No: ______________ Visit Date: (y/m/d) ___/___/___**

**Reviewer’s Initials: ­­­______**

**DATA COLLECTION FORM**

**Inclusion Criteria:**

1. Age >18? **Yes / No**
2. Patient came with anginal pain? **Yes / No**
3. Patient came with syncope **Yes? / No**
4. Patient came with heart failure symptoms **Yes? / No**

**Exclusion Criteria: (all below must be answered “NO” to continue)**

**1.** Patient known to have aortic valve replacement **Yes / No**

**2**-Patient known to have cardiac congenital anomalies other than bicuspid aortic valve? **Yes/ No**

**DOES PATIENT MEET ALL ABOVE CRITERIA? YES / NO**

**Demographics:**

1. Age: ______ 2. Gender: Male/Female

**PSLA View: Quality the of view: Poor(1) Adequate(2) Excellent(3)**

Presence of aortic valve calcification? Yes / No

**If Yes –** Mild/Moderate/Severe (circle one)

How many clearly visible aortic valve leaflets? None/One / Two/three

How many leaflets are moving? None/One / Two /three?

**PSSA View: Quality the of view: Poor(1) Adequate(2) Excellent(3)**

Presence of aortic valve calcification? Yes / No

**If Yes –** Mild/Moderate/Severe (circle one)

How many clearly visible aortic valve leaflets? None/One / Two/Three

How many leaflets are moving? None/One / Two/Three ?

**Conclusion:**

Is there severe aortic stenosis? Yes / No

Which view was the best to determine aortic valve assessment? **PSLA**  or **PSSA**

**Cardiology ECHO findings:**

**Quality of the view:**

**Poor(1) Adequate(2) Excellent(3)**

EF normal/abnormal

EF <45 Yes/No

**Conclusion**: Circle One

No AS, Mild AS/Moderate AS, Severe AS
